# Tumor necrosis factor-α-mediated threonine 435 phosphorylation of p65 nuclear factor-κB subunit in endothelial cells induces vasogenic edema and neutrophil infiltration in the rat piriform cortex following status epilepticus

**DOI:** 10.1186/1742-2094-9-6

**Published:** 2012-01-12

**Authors:** Ji-Eun Kim, Hea Jin Ryu, Soo Young Choi, Tae-Cheon Kang

**Affiliations:** 1 Department of Anatomy and Neurobiology, College of Medicine, Hallym University, Chunchon, Kangwon-Do 200-702, South Korea; 2 Institute of Epilepsy Research, College of Medicine, Hallym University, Chunchon, Kangwon-Do 200-702, South Korea; 3 Department of Biomedical Sciences, College of Life Science, Hallym University, Chunchon, Kangwon-Do 200-702, South Korea; 4 Department of Neurology, UCSF, and Veterans Affairs Medical Center, San Francisco, California 94121, USA

**Keywords:** Astrocyte, Blood brain barrier, Endothelium, Epilepsy, Immunohistochemistry

## Abstract

**Background:**

Status epilepticus (SE) induces severe vasogenic edema in the piriform cortex (PC) accompanied by neuronal and astroglial damages. To elucidate the mechanism of SE-induced vasogenic edema, we investigated the roles of tumor necrosis factor (TNF)-α in blood-brain barrier (BBB) disruption during vasogenic edema and its related events in rat epilepsy models provoked by pilocarpine-induced SE.

**Methods:**

SE was induced by pilocarpine in rats that were intracerebroventricularly infused with saline-, and soluble TNF p55 receptor (sTNFp55R) prior to SE induction. Thereafter, we performed Fluoro-Jade B staining and immunohistochemical studies for TNF-α and NF-κB subunits.

**Results:**

Following SE, most activated microglia showed strong TNF-α immunoreactivity. In addition, TNF p75 receptor expression was detected in endothelial cells as well as astrocytes. In addition, only p65-Thr435 phosphorylation was increased in endothelial cells accompanied by SMI-71 expression (an endothelial barrier antigen). Neutralization of TNF-α by soluble TNF p55 receptor (sTNFp55R) infusion attenuated SE-induced vasogenic edema and neuronal damages via inhibition of p65-Thr435 phosphorylation in endothelial cells. Furthermore, sTNFp55R infusion reduced SE-induced neutrophil infiltration in the PC.

**Conclusion:**

These findings suggest that impairments of endothelial cell functions via TNF-α-mediated p65-Thr 485 NF-κB phosphorylation may be involved in SE-induced vasogenic edema. Subsequently, vasogenic edema results in extensive neutrophil infiltration and neuronal-astroglial loss.

## Background

Status epilepticus (SE) is a medical emergency with significant mortality [1]. SE has been defined as continuous seizure activity, which causes neuronal cell death, epileptogenesis and learning impairment [2,3]. Some brain regions vulnerable to SE play a role in the generation and propagation of paroxysmal activity in experimental epilepsy models. The piriform cortex (PC) is one of the most susceptible brain regions to seizure-induced damage in the kainate, pilocarpine and other models of temporal lobe epilepsy (TLE) [4-6]. Pilocarpine, a cholinergic agonist, induces SE in rodents. This pilocarpine-induced SE, similar to human TLE, shows massive neuronal loss in the hippocampus followed by glial proliferation. This neuronal damage in the pilocarpine model is not restricted to the hippocampus, but often extends to extrahippocampal limbic structures. Indeed, pilocarpine-induced SE results in acute neuronal damages within layers II and III of the PC [5,6].

SE also induces severe vasogenic edema in the PC accompanied by neuronal and astroglial damages [5-8]. Brain edema proceeds in two phases, early cytotoxic edema phase and late vasogenic edema phase. Early cytotoxic osmotic edema is due to excess stimulation of glutamatergic pathways during SE, which increases intracellular Na^+ ^and Ca^2+ ^concentrations. The vasogenic edema results from dysfunction of endothelial cells and the blood-brain barrier (BBB). Many studies have reported increased permeability of the BBB during epileptic activity [9-13]. A fast and significant increase in systemic blood pressure, particularly shown during tonic epileptic seizures, induces a marked vasodilation of the large cerebral arteries and an increase in blood pressure in capillaries, small arteries, and veins leading to leakage of the BBB [9]. Loss of BBB integrity is not only due to an abrupt increase in the intraluminal pressure but also influenced by the properties of cerebral tissue. Indeed, an acute increase in blood pressure or epileptic activity causes an increase in pinocytosis at the level of the cerebral endothelium [11-13].

Recently, reports have also emphasized that seizure or epilepsy is a prolonged inflammatory condition, and that seizure activity rapidly increases the synthesis and release of various interleukins in rodent brain areas involved in seizure onset and their generalization. Cytokines act on endothelial cells and change the permeability of the BBB, which exerts significant effects on neuronal viability and excitability [14,15]. Indeed, Sztriha [16] reported that dexamethasone pretreatment reduces vasogenic edema in thalamus following kainic acid-induced seizure. Among cytokines, tumor necrosis factor-α (TNF-α) is a 17 kDa protein that is produced mainly by activated macrophages and T cells in the immune system. TNF-α is expressed at low levels in normal brain and is rapidly upregulated in glia, neurons and endothelial cells in various pathophysiological conditions, including SE [17,18]. TNF-α shows various effects on brain function depending on its local tissue concentration, the type of target cells, and especially the specific receptor subtype: TNF receptor I, or p55 receptor (TNFp55R); and TNF receptor II, or p75 receptor (TNFp75R) [19]. Furthermore, TNF-α induces macrophage inflammatory protein-2 (MIP-2) that recruits neutrophils under pathological conditions, including SE [14,20]. Neurons, microglia, and astrocytes produce MIP-2 when incubated with pro-inflammatory cytokines such as TNF-α and/or interleukin-1β (IL-1β) or after injury [21-23]. Indeed, we have recently reported that SE-mediated MIP-2 expression is relevant to leukocyte infiltrations following SE in an IL-1β-independent manner [20]. However, the relationship between the TNF-α system and BBB disruption/neutrophil infiltration during vasogenic edema formation induced by epileptogenic insults has not been fully clarified. Therefore, in the present study, we investigated the roles of TNF-α in vasogenic edema and its related events in rat epilepsy models provoked by pilocarpine-induced SE.

## Methods

### Experimental animals

This study utilized progeny of Sprague-Dawley (SD) rats (male, 9 - 11 weeks old) obtained from Experimental Animal Center, Hallym University, Chunchon, South Korea. The animals were provided with a commercial diet and water *ad libitum *under controlled temperature, humidity and lighting conditions (22 ± 2°C, 55 ± 5% and a 12:12 light/dark cycle with lights). Animal protocols were approved by the Institutional Animal Care and Use Committee of Hallym University. Procedures involving animals and their care were conducted in accord with our institutional guidelines that comply with NIH Guide for the Care and Use of Laboratory Animals (NIH Publications No. 80-23, 1996). In addition, we have made all efforts to minimize the number of animals used and their suffering.

### Intracerebroventricular drug infusion

Rats were divided into two groups: vehicle (saline)-treated and soluble TNFp55 receptor (sTNFp55R, 50 μg/ml; Sigma-Aldrich Co., St. Louis, MO)-treated groups. The dosage of sTNFp55R was determined as the highest dose that induced SE of comparable severity in 100% of animals with 5% mortality in the preliminary study. Animals were anesthetized (Zolretil, 50 mg/kg, i.m.; Virbac Laboratories, France) and placed in a stereotaxic frames. For the osmotic pump implantation, holes were drilled through the skull to introduce a brain infusion kit 1 (Alzet, Cupertino, CA) into the right lateral ventricle (1 mm posterior; 1.5 mm lateral; - 3.5 mm depth; flat skull position with bregma as reference), according to the atlas of Paxinos and Watson [24]. The infusion kit was sealed with dental cement and connected to an osmotic pump (1007D, Alzet, Cupertino, CA). The pump was placed in a subcutaneous pocket in the dorsal region. Animals received 0.5 μl/hr of vehicle or compound for 1 week. Therefore, the dose of sTNFp55R was 0.6 μg/day per each animal. The compounds began to be immediately infused after surgery. Since the volume of vasogenic edema peaked at 2-3 days after SE in our previous studies [5-8,20], we chose this time point. Thus, our experimental schedules at least inhibit the function of TNF-α from 3 days prior to SE to 4 days after SE when the volume of vasogenic edema peaked.

### Seizure induction

Three days after surgery, rats were treated with pilocarpine (380 mg/kg, i.p.; Sigma-Aldrich Co., St. Louis, MO) at 20 min after methylscopolamine (5 mg/kg, i.p.; Sigma-Aldrich Co., St. Louis, MO). Using this treatment paradigm, behavioral seizures typically began within 20-40 min. Approximately 80% of pilocarpine treated rats showed acute behavioral features of SE (including akinesia, facial automatisms, limbic seizures consisting of forelimb clonus with rearing, salivation, masticatory jaw movements, and falling). We applied the 2 hr-SE rat model, because > 90% of the rats that we monitored in our previous studies [25] displayed spontaneous, recurrent seizures within 1-3 months after pilocarpine-induced status epilepticus. Diazepam (10 mg/kg, i.p.; Hoffman Ia Roche, Neuilly sur-Seine) was administered 2 hours after onset of SE and repeated, as needed. The rats were then observed 3 - 4 hours a day in the vivarium for general behavior and occurrence of spontaneous seizures. Non-experienced SE (non-SE) rats (showing only acute seizure behaviors during 10 - 30 min, n = 8) and age-matched normal rats were used as controls (n = 7).

### Tissue processing

At designated time points (non-SE: 12 hr, 1 day, 2 days, 3 days, 4 days and 1 week after SE; n = 5, for each time point), animals were perfused transcardially with phosphate-buffered saline (PBS) followed by 4% paraformaldehyde in 0.1 M phosphate buffer (PB, pH 7.4) under urethane anesthesia (1.5 g/kg, i.p.; Sigma-Aldrich Co., St. Louis, MO). The brains were removed, and postfixed in the same fixative for 4 hr. The brain tissues were cryoprotected by infiltration with 30% sucrose overnight. Thereafter, the entire hippocampus was frozen and sectioned with a cryostat at 30 μm and consecutive sections were contained in six-well plates containing PBS. For stereological study, every sixth section in the series throughout the entire hippocampus was used in some animals.

### Immunohistochemistry

Free-floating sections were first incubated with 10% normal goat serum for 30 min at room temperature. They were then incubated in rabbit anti-MPO IgG (1:100, Thermo fisher scientific) or rabbit anti-MIP-2 IgG (1:200, Invitrogen, Carlsbad, CA) in PBS containing 0.3% Triton X-100 (Sigma-Aldrich Co., St. Louis, MO) and 2% normal goat serum(Sigma-Aldrich Co., St. Louis, MO) overnight at room temperature. After washing three times for 10 min with PBS, the sections were incubated sequentially, in goat anti-rabbit or horse anti-mouse IgG (Vector, Burlingame, CA) and ABC complex (Vector, Burlingame, CA), diluted 1:200 in the same solution as the primary antiserum. Between the incubations, the tissues were washed with PBS three times for 10 min each. To confirm vasogenic edema, some tissue sections were reacted for serum-proteins using horse anti-rat IgG (Vector, Burlingame, CA) as a primary antibody. The sections were visualized with 3,3'-diaminobenzidine (DAB, Sigma-Aldrich Co., St. Louis, MO) in 0.1 M Tris buffer and mounted on the gelatin-coated slides. The immunoreactions were observed under the Axioscope microscope (Carl Zeiss, Munchen-Hallbergmoos). For negative controls, rat hippocampal tissues were incubated with 1 μg of the antibody that was preincubated with 1 μg of purified peptide for 1 hr at room temperature or incubated with pre-immune serum instead of the primary antibody. For negative controls, tissues were incubated with pre-immune serum instead of primary antibody.

### Double immunofluorescence study

Sections were incubated with 3% bovine serum albumin in PBS for 30 min at room temperature. Sections were then incubated in a mixture of goat anti-TNF-α IgG (1:1000, R&D systems, Minneapolis, MN)/mouse anti-OX-42 IgG (1:100, Serotec, Cambridge, UK), mouse anti-GFAP IgG (1:1000, an astroglial marker, Millipore Corporation, Billerica, MA)/rabbit anti-TNFp55R IgG (1:1000, Abcam, Cambridge, UK), mouse anti-GFAP IgG/rabbit anti-TNFp75R IgG (1:1000, Abcam, Cambridge, UK), mouse anti-SMI-71 IgM (1:1000, Covance, Berkeley, CA)/rabbit anti-TNFp75R IgG, mouse anti-GFAP IgG/rabbit anti-NF-κB (p65-Ser276, p65-Ser311, p65-Ser529, and p65-Thr435) IgG (1:100, Abcam, Cambridge, UK), mouse anti-SMI-71 IgM/rabbit anti-p65-Thr435 NF-κB IgG, mouse anti-SMI-71 IgM/rabbit anti-GLUT-1 IgG (1:100, Abcam, Cambridge, UK), or mouse anti-GFAP IgG/rabbit anti-MIP-2 IgG (1:100) in PBS containing 0.3% triton X-100 overnight at room temperature. After washing three times for 10 minutes with PBS, sections were also incubated in a mixture of FITC- and Cy3-conjugated secondary antisera (Amersham, San Francisco, CA), diluted 1:200, for 2 hr at room temperature. The sections were washed three times for 10 min with PBS, and mounted on gelatin-coated slides. For nuclei counterstaining, we used Vectashield mounting medium with DAPI (Vector, Burlingame, CA). All images were captured using an AxioImage M2 microscope and AxioVision Rel. 4.8 software.

### Fluoro-Jade B staining

Fluoro-Jade B (FJB) staining was used to identify degenerating neurons in tissues obtained from non-SE and 3 days post-SE animals in every group. In our previous [18,25] and preliminary data, neuronal damage was first detectable at 3 days after SE. Therefore, we determined 3 days after SE as the best time point to look FJB. Briefly, sections were rinsed in distilled water, and mounted onto gelatin-coated slides and then dried on a slide warmer. The slides were immersed in 100% ethanol for 3 min, followed by 70% ethanol for 2 min and distilled water for 2 min. The slides were then transferred to 0.06% potassium permanganate for 15 min and gently agitated. After rinsing in distilled water for 2 min, the slides were incubated for 30 min in 0.001% FJB (Histo-Chem Inc., Jefferson, AR), freshly prepared by adding 20 ml of a 0.01% stock FJB solution to 180 ml of 0.1% acetic acid, with gentle shaking in the dark. After rinsing for 1 min in each of three changes of distilled water, the slides were dried, dehydrated in xylene and coverslipped with DPX (Sigma-Aldrich Co., St. Louis, MO). For stereological study, every sixth section in the series throughout the entire PC was used (see below).

### Volumetric analysis and cell counts

To measure vasogenic edema, the volume of anti-rat IgG positive region in PC was estimated according to the formula based on the modified Cavalieri method: V = *Σa *× *t*_nom _× 1/ssf, where *a *is area of the region of the delineated subfield measured by AxioVision Rel. 4.8 software, *t*_nom _is the nominal section thickness (of 30 μm in this study), and ssf is the fraction of the sections sampled or section sampling fraction (of 1/6 in this study). The subfield areas were delineated with a 2.5 × objective lens [5,7,8,18,25]. The volumes are reported as mm^3^. An optical fractionator was used to estimate cell numbers. The optical fractionator (a combination of performing counting with the optical dissector, with fractionator sampling) is a stereological method based on a properly designed systematic random sampling method that by definition yields unbiased estimates of population number. The sampling procedure is accomplished by focusing through the depth of the tissue (the optical dissector height, h; 15 μm in all cases for this study). The number of each cell type (C) in each of the subregions is estimated as: C = *ΣQ^- ^*× t/h × 1/asf × 1/ssf, where *Q^- ^*is the number of cells actually counted in the dissectors that fell within the sectional profiles of the subregion seen on the sampled sections, and Asf is the areal sampling fraction calculated by the area of the counting frame of the dissector, a(frame) (of 50 × 50 μm^2 ^in this study) and the area associated with each x, y movement, grid (x, y step) (of 250 × 250 μm^2 ^in this study) {asf = (a(frame)/a(x, y step))}. The immunoreactive cells were counted with a 40× objective lens. The immunoreactive cells were counted with a 40× objective lens. All immunoreactive cells were counted regardless the intensity of labeling. Cell counts were performed by two different investigators who were blind to the classification of tissues. SE-induced PC atrophy is evident [8], so changes in cell number may be caused by an alterations in the volume of the PC. Therefore, the total number of cells was corrected by multiplying with appropriate correction factors (CF) representing the degree of shrinkage (or swelling) compared with the Non-SE.

### Quantification of data

The fluorescence intensities of SMI-71/p65-Thr435 phosphorylation or GFPA/p65-Thr435 phosphorylation were measured using a computer-assisted image analysis program (The University of Texas ImageTool program V. 3.0 and AxioVision Rel. 4.8 software). After regions were outlined, 30 areas/rat (300 μm^2^/area) were randomly selected within the PC, and double immunofluorescent merge images were captured from the PC (15 sections from each animal). Merge images were digitally separated to red or green image, and converted to grayscale images, respectively (n = 36 per region examined, in non-SE, 12 hr post-SE and 1 day post-SE). The range of intensity values was obtained from the selected images. Based on the mean range of intensity values, each image was normalized by adjusting the black and white range of the image. Manipulation of the images was restricted to threshold and brightness adjustments to the whole image. Intensity measurements are represented as the mean number of a 256 gray scale (NIH Image 1.59 software and AxioVision Rel. 4.8 software). Values for background staining were obtained from the corpus callosum. Optical density values were then corrected by subtracting the average values of background noise obtained from 15 image inputs.

### Data analysis

Data obtained from volumetric analysis, cell counts, and quantitative measurements were analyzed using Student's *t*-test to determine statistical significance. Linear regression analysis was also performed to determine correlations with SMI-71/p65-Thr435 phosphorylation, and the number of MPO cells/vasogenic edema areas.

## Results

### TNF-α, and TNF receptor expression

**Figure 1:**
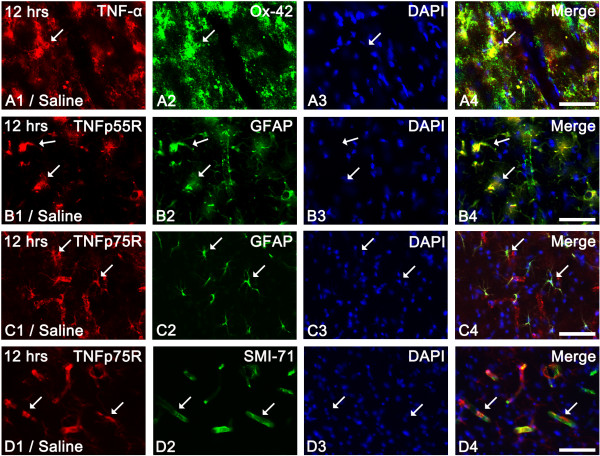
**Expression of TNF-α and TNF receptor in the PC 12 hr-post SE**. (A) TNF-α immunoreactivity in Ox-42-positive microglia (arrows). (**B**) TNFp55R expression in astrocytes (arrows). (**C-D**) TNFp75R expression in astrocytes as well as endothelial cells (arrows). Bar = 25 (**A-D**) μm.

In non-SE induced animals of the saline-infused groups, TNF-α immunoreactivity was weakly detected in PC neurons (data not shown). In 12 hr-post SE animals of the saline-infused group, most of the activated microglia showed strong TNF-α immunoreactivity (Figure 1A). This expression pattern was maintained up to 1 week after SE. In non-SE-induced animals of the saline-infused groups, TNFp55R and TNFp75R immunoreactivities were also weakly observed in astrocytes (data not shown). In 12 hr-post SE animals of the saline-infused group, TNFp55R immunoreactivity was observed in astrocytes (Figure 1B). Unlike TNFp55R, TNFp75R immunoreactivity was detected in endothelial cells as well as astrocytes (Figures 1C-E). One day to 1 week after SE, both TNFp55R and TNFp75R immunoreactivities were significantly reduced in astrocytes, not in endothelial cells, due to massive astroglial loss (data not shown) [5,7,8,20].

### Effect of sTNFp55R infusion on SE-induced serum-protein extravasation and neuronal damage

**Figure 2:**
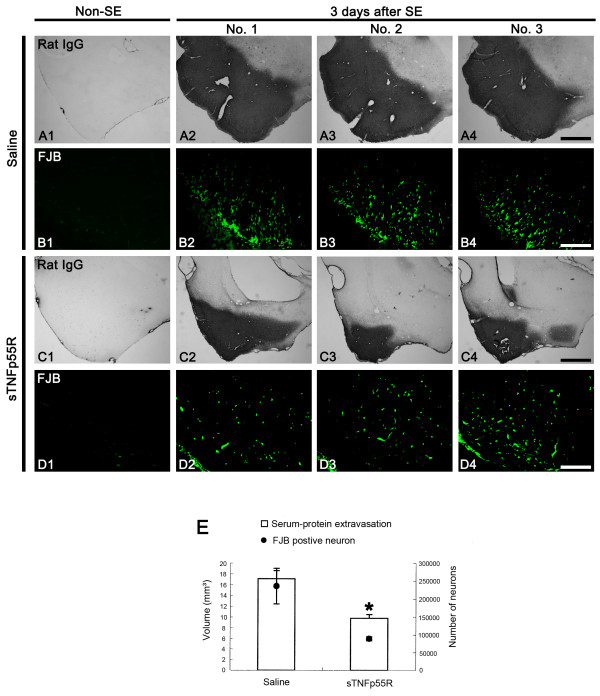
**Effect of sTNFp55R infusion on SE-induced serum-protein extravasation and neuronal damage**. (**A-D**) Serum-protein extravasation and FJB-positive neuronal damages in the PC 3 days after SE. Compared to saline-infused animals, serum-protein extravasation and FJB-positive neuronal damage is markedly ameliorated in sTNFp55R-infused animals. Bars = 400 (**A **and **C**) and 50 (**B **and **D**) μm. (**E**) Quantitative analyses of serum-protein extravasation and FJB-positive neuronal damage in the PC 3 days after SE (mean ± S.E.M). Significant differences from saline-treated animals, *p < 0.05.

In our previous [5,7,8,20] and preliminary data, vasogenic edema and neuronal damage were noticeable at 1 day and 3 days after **SE, respectively**. Therefore, we determined that 3 days after **SE **was the best time point to evaluate the effect of sTNFp55R infusion on both vasogenic edema and neuronal damages induced by SE. In saline-treated animals, the PC was stained diffusely with anti-rat IgG (Figure 2A). The volume of vasogenic edema was 17.1 ± 1.5 mm^3 ^(Figure 2E). The number of FJB-positive neurons in the PC was 236,145 ± 49,469 (Figure 2B). In sTNFp55R-treated animals, SE-induced vasogenic edema was attenuated to 9.8 ± 0.7 mm^3 ^(Figures 2C and 2E). In addition, the number of FJB-positive neurons in the PC was 89,138 ± 5,698 (Figures 2D-E). Thus, sTNFp55R infusion attenuated SE-induced vasogenic edema and neuronal damage compared to saline-infused animals (p < 0.05).

### NF-κB phosphorylation

**Figure 3:**
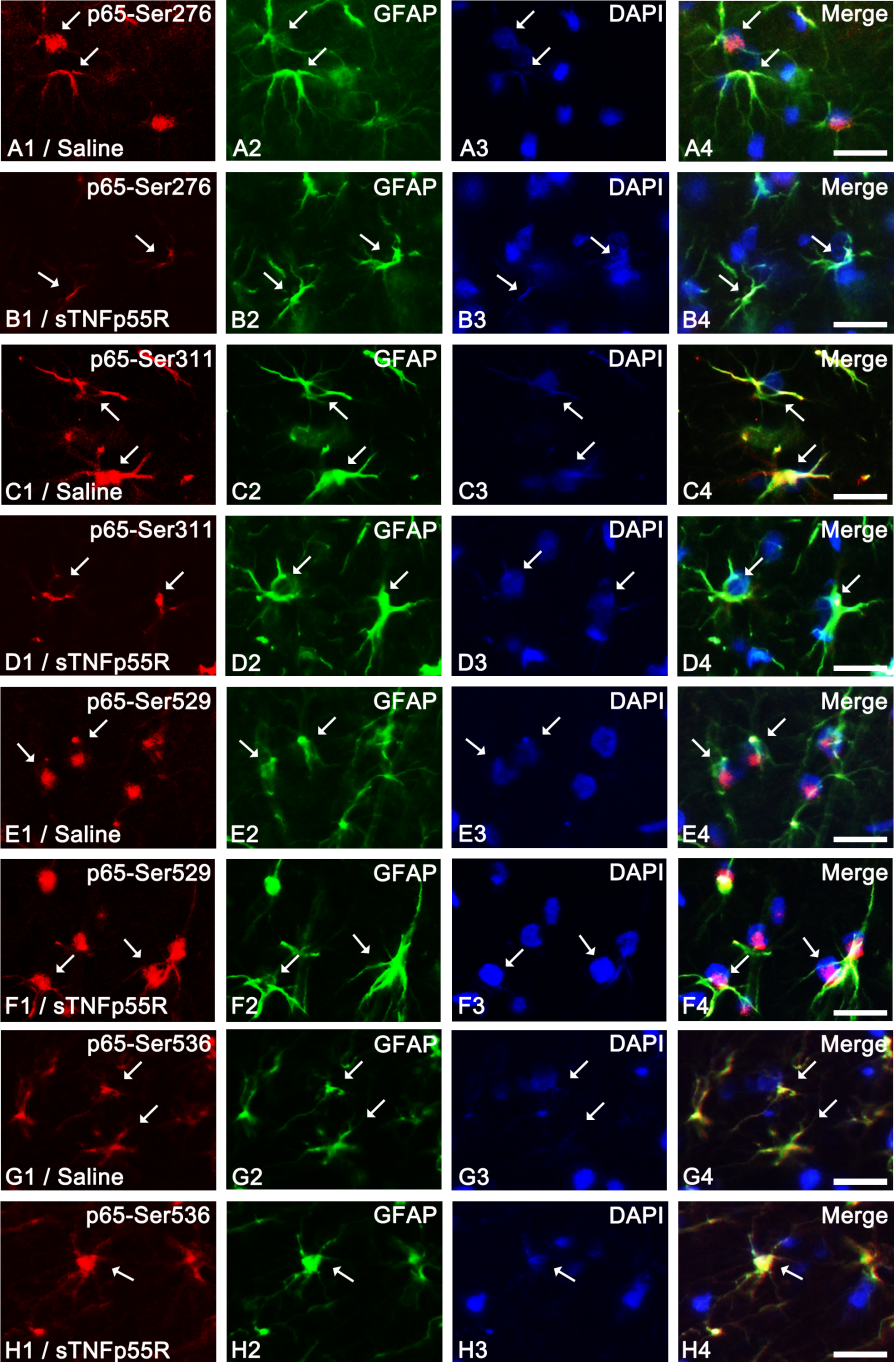
**Effect of sTNFp55R infusion on NF-κB phosphorylation in astrocytes 12 hr after SE**. In 12 hr-post SE animals of the saline-infused group (**A**, **C**, **E **and **G**), astrocytes show p65-Ser276 (**A**), p65-Ser311 (**C**), p65-Ser529 (**E**), and p65-Ser536 (**G**) phosphorylation (arrows). sTNFp55R infusion (**B**, **D**, **F **and **H**) effectively reduces p65-Ser276 (**B**) and p65-Ser311 (**D**) phosphorylation, while it does not affect p65-Ser529 (**F**) and p65-Ser536 (**H**) phosphorylation (arrows). Bar = 12.5 μm.

**Figure 4:**
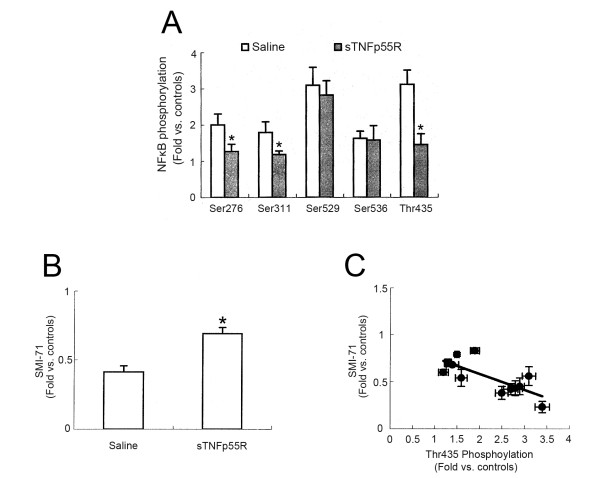
**Quantitative analyses of the effect of sTNFp55R infusion on NF-κB phosphorylation and SMI-71 expression**. (**A**) Quantitative analysis of NF-κB phosphorylation 12 hr after SE (mean ± S.E.M). Significant differences from saline-infused animals, *p < 0.05. (**B**) Quantitative analysis of SMI-71 expression 1 day after SE (mean ± S.E.M). Significant differences from saline-infused animals, *p < 0.05. (**C**) Linear regression analysis between p65-Thr435 phosphorylation and SMI-71 in the PC following SE.

**Figure 5:**
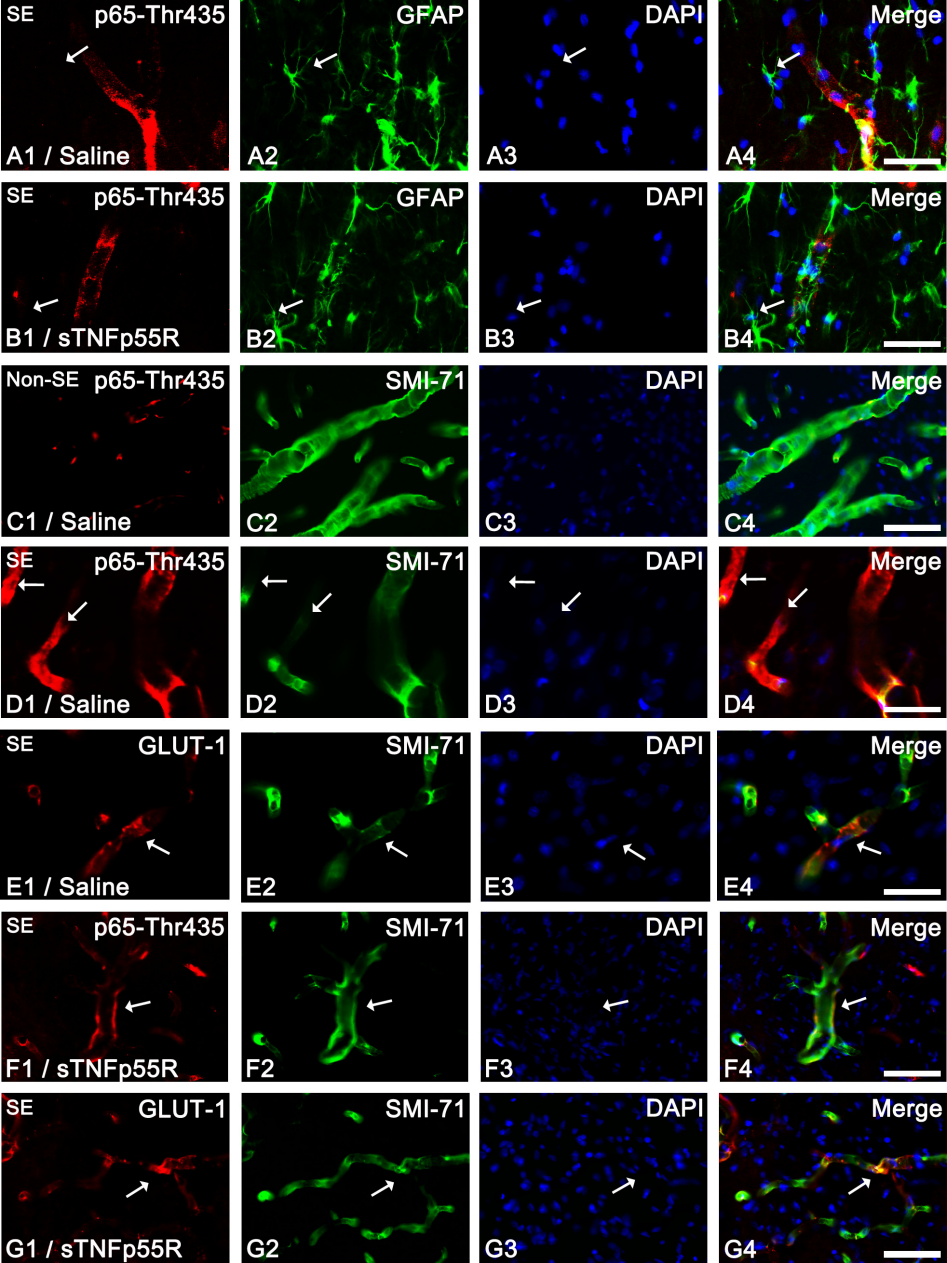
**Effect of sTNFp55R infusion on p65-Thr435 phosphorylation in endothelial cells following SE**. (**A-B) **Inhibition of p65-Thr435 phosphorylation by sTNFp55R infusion 12 hr after SE. p65-Thr435 phosphorylation is rarely observed in astrocytes (arrows). (**C**) Endothelial p65-Thr435 phosphorylation in non-SE animals. (**D-E**) Endothelial p65-Thr435 phosphorylation in saline-infused animals 1 day after SE. p65-Thr435 phosphorylation is enhanced, while SMI-71 expression is reduced in GLUT-1-positive endothelial cells (arrows). (**F-G**) Endothelial p65-Thr435 phosphorylation in sTNFp55R-infused animal 1 day after SE. sTNFp55R infusion effectively reduces p65-Thr435 and preserves SMI-71 expression in GLUT-1-positive endothelial cells (arrows). Bars = 12.5 (**A-D**) and 25 (**E-G**) μm.

It is well established that TNF-α is one of the major stimuli toward phosphorylation of NF-κB. To confirm TNF-α-mediated signaling following SE, we performed an immunohistochemical study using five phospho-NF-κB antibodies. Compared to non-SE animals (data not shown), 12 hr-post SE animals of the saline-infused group showed p65-Ser276, p65-Ser311, p65-Ser529, and p65-Ser536 phosphorylation in astrocytes (not endothelial cells). sTNFp55R infusion effectively reduced p65-Ser276 and p65-Ser311 phosphorylation (p < 0.05, respectively), while it could not affect p65-Ser529 or p65-Ser536 phosphorylation (Figures 3 and 4A). In contrast, p65-Thr435 phosphorylation was increased in endothelial cells (not astrocytes) within the PC of saline-infused animals 12 hr after SE (Figure 5A). In addition, sTNFp55R infusion effectively alleviated SE-induced p65-Thr435 phosphorylation in endothelial cells, compared to saline infusion (p < 0.05, Figure 5B).

### SMI-71 expression

Previously, we reported that SMI-71 (an endothelial barrier antigen) immunoreactivity decreased in the PC 1 day after SE [5]. Similarly, in 1 day-post SE animals of the saline-infused group, loss of SMI-71 immunoreactivity was detected in layer III/IV of the PC as compared to non-SE animals (Figures 4B and 5C-E, p < 0.05). Thus, loss of SMI-71 immunoreactivity correlated with volume of vasogenic edema following SE. This reduction in SMI-71 was accompanied by increased p65-Thr435 phosphorylation (Figures 5C-D). Therefore, the degree of SMI-71 immunoreactivity was inversely correlated to p65-Thr435 phosphorylation with a linear coefficient of correlation of -0.6324 (p < 0.05; Figure 4C). In addition, sTNFp55R infusion effectively alleviated p65-Thr435 phosphorylation and preserved SMI-71 immunoreactivity in endothelial cells following SE, as compared to saline infusion (p < 0.05; Figures 4A-B, 5D and 5F).

### Neutrophil infiltration

**Figure 6:**
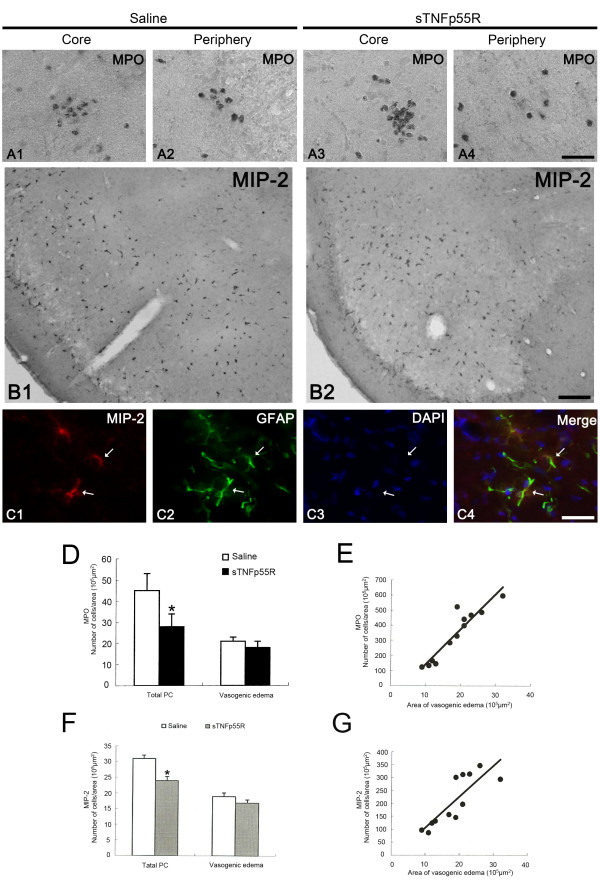
**Effect of sTNFp55R infusion on neutrophil infiltration and MIP-2 expression following SE**. (**A**) Neutrophil infiltration in vasogenic edema lesion 1 day after SE. (**B**) MIP-2 expression in the PC 1 day after SE. (**C**) Astroglial expression of MIP-2 (arrows). Bars = 12.5 (**A **and **C**) and 150 (**B**) μm. (**D**) Quantitative analysis of neutrophil infiltration 1 day after SE (mean ± S.E.M). Significant differences from saline-infused animals, *p < 0.05. (**E**) Linear regression analysis between the number of infiltrated neutrophils/area in the vasogenic edema region and the area of vasogenic edema in the PC. (**F**) Quantitative analysis of the number of MIP-2 positive cells per the unit area of vasogenic edema 1 day after SE (mean ± S.E.M). There is no difference in the number of MIP-2-positive cells per unit area of vasogenic edema between sTNFp55R-infused animals and saline-infused animals. (**G**) Linear regression analysis between the number of MIP-2 positive cells per unit area in vasogenic edema region and the area of vasogenic edema in the PC.

Recent studies have reported that neutrophils infiltrate the brain under certain pathological conditions [26]. Indeed, we have reported massive neutrophil infiltration in layer III/IV of the PC 1 day after SE [20]. In the present study, 1 day-post-SE animals of the saline-infused group showed infiltration of MPO-positive neutrophils into the PC. Similarly, 1 day-post-SE animals of the sTNFp55R-infused group showed neutrophil infiltration into the PC 1 day after SE (Figure 6A). The number of neutrophils/area in the PC region (including the vasogenic edema region and the non-vasogenic edema region) of sTNFp55R-infused animals was significantly lower than that of the saline-infused group (Figure 6D, p < 0.05). However, there was no difference in neutrophil infiltration per unit area of vasogenic edema between the saline- and sTNFp55R-infused groups (Figure 6D). Furthermore, neutrophil infiltration showed a direct proportion to the area of vasogenic edema, with a linear coefficient of correlation of 0.8631 (p < 0.05, Figure 6E). Therefore, our findings indicate that SE-induced neutrophil infiltration into the PC may be correlated to TNF-α-mediated vasogenic edema formation.

### MIP-2 expression

MIP-2 is a powerful chemokine that contributes to recruitment of neutrophils [27]. MIP-2 is undetectable or present at low levels under physiological conditions, and shows transient increases under pathological conditions via TNF-α and/or interleukin-1β (IL-1β)-dependent mechanisms [14]. Thus, it would be plausible that TNF-α-mediated MIP-2 expression may provoke SE-induced neutrophil infiltrations. To confirm this hypothesis, we investigated MIP-2 expression in the PC. Consistent with our previous study [20], some MIP-2-positive astrocytes were observed in the core and periphery of the vasogenic edema lesions, but not in the non-vasogenic edema region (Figure 6B and 6C). Although the number of MIP-2 positive cells per unit area in the PC region of sTNFp55R-infused animals was significantly lower than that of the saline-infused group due to reduction of the area of vasogenic edema, there was no difference in the number of MIP-2 positive cells per unit area of vasogenic edema between sTNFp55R-infused animals and saline-infused animals (Figure 6F). Furthermore, the number of MIP-2-positive cells showed a direct proportion to the unit area of vasogenic edema with a linear coefficient of correlation of 0.682 (p < 0.05, Figure 6G). Therefore, together with reduction in neutrophil infiltration in the PC region of sTNFp55R-infused animals, our findings provide evidence that TNF-α may regulate SE-induced neutrophil infiltration at least in the PC via vasogenic edema formation and not via direct TNF-α-mediated MIP-2 expression in astrocytes.

## Discussion

The major findings in the present study are that TNF-α signaling showed cellular specific responses of NF-κB phosphorylation in the PC following SE, which may be related to vasogenic edema formation followed by neutrophil infiltration. BBB disruption has been reported in experimental and human epilepsy [12,13,15,16,28]. Leakage of serum-derived components into the extracellular space is associated with hyperexcitability and seizure onset [12,13,15,16,28]. Furthermore, dysfunction of the BBB leads to epileptogenesis and contributes to progression of epilepsy [12,13,15,16,28]. In the present study, TNF-α immunoreactivity was obviously observed in microglia in the PC following SE. TNF receptor expressions were also up-regulated in astrocytes (TNFp55R and TNFp75R) and endothelial cells (TNFp75R). Furthermore, blockade of TNF-α signaling by sTNFp55 infusion effectively (but not completely) reduced volumes of SE-induced vasogenic edema and neuronal damage in the PC. These findings indicate that TNF-α may participate in astroglial and endothelial responses to SE, which are relevant to SE-induced vasogenic edema formation [5-8]. Indeed, TNF-α signaling increases BBB permeability in various experimental disease models [29]. In the present study, sTNFp55 infusion could not completely prevent SE-induced vasogenic edema and neuronal damage in the PC. Therefore, our findings suggest that TNF-α signaling may not be a unique upstream event in vasogenic edema development.

p65 phosphorylation of NF-κB enhances its transactivation potential, and p65 phosphorylation occurs in either the cytoplasm or the nucleus [30]. In the present study, p65-Thr435 immunoreactivity was detected in endothelial cells, and its immunoreactivity showed an inverse correlation to the degree of SMI-71 expression. SMI-71, an endothelial barrier antigen, is a protein expressed by endothelial cells of rat BBB [31]. Under pathological conditions, SMI-71 expression is lost in endothelial cells [5,7,8,30,32]. Acute phases of the above pathological conditions are accompanied by opening of the BBB and development of vasogenic edema [33]. Indeed, neutralization of SMI-71 *in vivo *leads to widening of intercellular junctions between endothelial cells and swelling of perivascular astrocytic processes [34], although SMI-71 is not localized at endothelial cell junctions [35-38]. In the present study, SMI-71 immunoreactivity was significantly reduced in blood vessels 1 day after SE when vasogenic edema and neuronal damage were observed. Furthermore, sTNFp55R infusion effectively prevented SE-induced SMI-71 down-regulation. With respect to the phosphorylation of p65-Thr435 by TNF-α [39], our findings indicate that TNF-α-mediated p65-Thr435 phosphorylation in endothelial cells may play an important role in vasogenic edema induction via SMI-71 degradation or its posttranslational dysfunction influencing BBB permeability.

In our previous studies [5,8], dystrophin (an actin-binding protein [40]) immunoreactivity was detected in blood vessels and in astrocytic perivascular end-feet, and was down-regulated 12 hrs after SE prior to the appearance of vasogenic edema and down-regulation of SMI-71 immunoreactivity. With respect to this previous report, changes in SMI immunoreactivity would be causes/results of interaction between endothelial cells and perivascular astrocytes. In the present study, p65-Ser276, p65-Ser311, p65-Ser529, and p65-Ser536 phosphorylation was observed in astrocytes following SE. Furthermore, sTNFp55R infusion effectively inhibited p65-Ser276 and p65-Ser311phosphorylation in astrocytes following SE. Therefore, it is likely that enhanced p65-Ser276 and p65-Ser311 phosphorylation may be involved in TNF-α-mediated BBB disruption. However, sTNFp55R infusion could not prevent p65-Ser529 and p65-Ser536 phosphorylations from SE insults. Since p65-Ser529 and p65-Ser536 are phosphorylated by TNF-α and IL-1β [41], it is likely that IL-1β-mediated p65-Ser529/Ser536 phosphorylation may also play a role in SE-induced vasogenic edema. Therefore, our findings indicate that both TNF-α and IL-1β may be synergists to play either a direct (by endothelial cells) or indirect (by astrocytes) role in the maintenance of BBB permeability.

Neutrophil infiltration into brain parenchyma is transiently observed during the acute phase of SE (4 - 36 hr after SE) and disappears thereafter [20]. SE rapidly increases synthesis and release of chemokines in various areas of the rodent brain [42]. Among them, MIP-2 is required for efficient neutrophil or lymphocyte recruitment to brain parenchyma [43]. In our previous study [20], neutrophil infiltration in the frontoparietal cortex was regulated by P2X7 receptor-mediated MIP-2 expression. In the PC, however, neither a P2X7 receptor agonist/antagonist nor IL-1Ra (an IL-1β antagonist) infusion could not affect leukocyte infiltration. In the present study, sTNFp55R infusion effectively inhibited neutrophil infiltration in the PC by reducing vasogenic edema formation in a MIP-2-independent manner. With respect to the present and our previous reports, it is therefore likely that vasogenic edema induced by TNF-α can induce neutrophil infiltration and press injury to evoke neuronal-astroglial loss in the PC, unlike other brain regions.

In conclusion, our findings reveal that impairments of endothelial cell function via TNF-α mediated p65-Thr 435 NF-κB phosphorylation may be involved in SE-induced vasogenic edema, which is relevant to neutrophil infiltration and neuronal-astroglial loss.

## Competing interests

The authors declare that they have no competing interests.

## Authors' contributions

JEK and HJR were involved in designing and performing all experiments. SYC and TCK helped in drafting the manuscript. JEK and HJR did the immunohistochemistry, the intracerebroventricular drug infusion, the seizure studies and the acquisition of data and analyses. SYC and TCK provided continuous intellectual input, and evaluation and interpretation of data. All authors read and approved the final manuscript.
